# CSF and Serum Biomarkers of Cerebral Damage in Autoimmune Epilepsy

**DOI:** 10.3389/fneur.2021.647428

**Published:** 2021-04-16

**Authors:** Robert Daniel Nass, Katja Akgün, Karmele Olaciregui Dague, Christian Erich Elger, Heinz Reichmann, Tjalf Ziemssen, Rainer Surges

**Affiliations:** ^1^Department of Epileptology, University Hospital Bonn, Bonn, Germany; ^2^Department of Neurology, Dresden University Hospital, Dresden, Germany

**Keywords:** CSF, serum, epilepsy, tau, neurofilament, NFL, UCHL-1, GFAP

## Abstract

**Introduction:** Our goal was to investigate whether biomarkers of cerebral damage are found in autoimmune-mediated epilepsy (AIE) and whether these can differentiate AIE from other seizure disorders.

**Methods:** We retrospectively searched our cerebrospinal fluid (CSF) database for patients with definite AIE, hippocampal sclerosis due to other causes (HS), genetic generalized epilepsy (GGE), and psychogenic, non-epileptic seizures (PNES). We measured serum and CSF tau, neurofilament 1 (NFL), glial fibrillary acid protein (GFAP), and ubiquitin-carboxy-terminal hydrolase L1 with a single-molecule array.

**Results:** We identified suitable samples from patients with AIE (*n* = 13) with different antibodies and compared them to HS (*n* = 13), GGE (*n* = 7), and PNES (*n* = 8). The NFL levels were significantly elevated in the serum (*p* = 0.0009) and CSF (*p* < 0.0019) of AIE patients. The AIE group was significantly older, while the disease duration was significantly shorter than in the control groups. NFL correlated significantly with age in all groups, and the NFL levels of AIE patients were hardly higher than those of healthy elderly people published elsewhere.

**Conclusions:** Our data indicate that the elevated NFL levels in AIE patients are most likely due to the higher age in this group and not due to the underlying inflammation. Unless larger prospective studies with intra-individual, longitudinal analyses and treatment responses would contradict our findings, NFL in serum might yet become a biomarker for disease activity and differential diagnosis.

## Introduction

In the past two decades, a subset of epilepsies, particularly temporal lobe epilepsy (TLE), has been recognized as an antibody-mediated autoimmune disorder. The most common phenotypes are non-paraneoplastic and paraneoplastic limbic encephalitis (1). The diagnostic criteria (2) include subacute onset seizures, neurocognitive changes, cerebral magnetic resonance imaging changes suggestive of encephalitis with FLAIR hyperintensities of the temporal lobes and/or other cortical areas, and cerebrospinal fluid (CSF) pleocytosis. The presence of autoantibodies is not mandatory for making the diagnosis if all criteria are present. Antibody testing is important in cases that do not meet all criteria in order to define the clinical syndrome and to plan the appropriate short- and long-term treatments (2). The histopathological correlates in brain biopsies reach from mostly lymphocyte-driven inflammation of limbic structures to hippocampal sclerosis (HS) (3).

The ever-growing list of autoantibodies (ABs) include “onconeural” antibodies such as amphiphysin, BMP-binding endothelial regulator (anti-BMPER and anti-CV2), paraneoplastic Ma antigen 2 (anti-Ma2 and anti-PNMA2), and anti–glutamic acid decarboxylase 65 (anti-GAD65), all of them aimed against intracellular protein structures. ABs which target the neuronal cell membrane proteins include N-methyl-D-aspartate receptors (NMDAR), leucine-rich glioma inactivated 1 (LGI1), contactin-associated protein 2 (CASPR2), α-amino-3-hydroxy-5-methyl-4-isoxazolepropionic acid receptors (AMPAR), γ-aminobutyric acid receptor A and B (GABAR), dipeptidyl-peptidase-like protein-6, and metabotropic glutamate receptor 5 (2). More recent additions include neuronal surface glycine receptors (4) and cytoplasmatic drebrin (5) and Zic4 (6).

In autoimmune-mediated epilepsy (AIE), it is important not only to treat the seizures but also the underlying autoimmune encephalitis (7) with immunomodulatory therapies, i.e., steroids, immunoglobulins, plasma exchange, immunoabsorption, rituximab, cyclophosphamide, or other agents.

The main markers to assess the disease course are seizures, neurocognitive alterations, MRI changes, CSF normalization, and antibody titers. Furthermore, easily available biomarkers would be desirable to investigate whether ongoing neuronal damage due to active inflammation occurring in an individual AIE patient may indicate disease progression or relapse or could help to determine when to stop immunosuppressive therapy.

In recent years, ultra-high-sensitive serum and CSF measurements for markers of neuronal and glial cellular damage have become available and have been studied in a range of neuroimmunological disorders (8), including autoimmune encephalitis (9–12).

Here we investigated whether markers of neural damage can distinguish between autoimmune phenomena and other types of epilepsy.

## Subjects and Methods

We retrospectively searched our CSF and serum database (03/2015–06/2018) for patients with AIE, HS due to other causes (HS), genetic generalized epilepsy (GGE), and psychogenic, non-epileptic seizures (PNES). Same day serum and CSF samples were in storage at −80°C.

The patients had initially received their lumbar puncture (LP) to rule out inflammatory epilepsy because there were hints toward that diagnosis such as new-onset epilepsy, cognitive impairment, signs of temporal lobe onset on initial EEG recordings, asymmetries between the hippocampi or amygdalae on MRI, or other features that suggested possible AIE. Only patients that fulfilled the definite criteria of PNES, GGE, HS, or AIE were included. Probable and possible cases as well as cases with a double diagnosis, e.g., PNES + HS were excluded. PNES patients had definite non-epileptic events documented on video—EEG without additional signs of epilepsy or brain pathology. Patients with GGE had reported generalized seizure types (generalized absence seizure, GAS; generalized myoclonic seizures, GMS; generalized tonic clonic seizures, GTCS; or mixed generalized seizures, i.e., GTCS + GAS or GTCS + GMS). They had generalized epileptiform potentials on video EEG and no clear MRI or laboratory anomalies that would have suggested another cause of their epilepsy. Patients with HS had clear HS on MRI and, in some cases, on histopathology. Patients with AIE had a limbic syndrome with behavioral and cognitive changes as well as temporal lobe EEG changes or seizures and MRI alterations of their mesial temporal lobes. AIE was confirmed in all cases with positive antibodies. Patients were excluded from the PNES, GGE, and HS groups if they had low titer, non-replicable autoantibodies, or other active neurological diseases. If patients with AIE had received immunotherapy prior to their LP, they were excluded.

We measured tau, neurofilament 1 (NFL), glial fibrillary acid protein (GFAP), and ubiquitin-carboxy-terminal hydrolase L1 (UCHL1) with a highly sensitive single-molecule array (SIMOA® by Quanterix®) with the manufacturer's Neurology 4-plex panel®. The data were examined using Kruskall–Wallis test and Dunn's post-test as well as receiver operating characteristic (ROC) analysis. The data are reported as median and interquartile range.

The study was approved by the Ethical Commission of the University Hospital Bonn as part of a larger, long-term project on autoimmune phenomena in epilepsy (222/16).

## Results

At the time of the study measurements (June 2018), the database contained the serum and CSF of 520 patients. All patients were under anti-epileptic treatment at the time of their LP. The exclusion process yielded 41 patients: 13 AIE with different antibodies (6xLGI-1, 4xCASPR2, 1xNMDAR, 1xGAD65, and 1xZic), 13 HS, seven GGE, and eight PNES. The clinical and demographic characteristics of the four patient groups are summarized in Tables 1, 2.

**Table 1:** Clinical characteristics (categorical).

		**PNES (*n* = 8)**		**GGE (*n* = 7)**		**HS (*n* = 13)**		**AIE (*n* = 13)**		**Significance**
	***N***	**%**	***N***	**%**	***N***	**%**	***N***	**%**	**χ^2^**	
Sex	Female	6	75	3	42.9	6	46.2	4	36.4	n.s.
	Male	2	25	4	57.1	7	53.8	7	63.6	
Seizure types	PNES	8	100	0	0	0	0	0	0	*p* < 0.0001
	GAS	0	0	1	14.2	0	0	0	0	
	GMS	0	0	1	14.2	0	0	0	0	
	GTCS	0	0	7	100	0	0	0	0	
	FS	0	0	0	0	5	38.5	8	61.5	
	FTBTCS	0	0	0	0	8	61.5	5	38.5	
Cognition	Intact	4	50	5	71.4	0	0	2	15.3	*p* = 0.002
	Altered	4	50	2	28.5	13	100	11	84.6	
Emotion	Intact	2	25	3	42.9	3	23.1	5	38.5	n.s.
	Altered	6	75	4	57.1	10	76.9	8	61.5	
BCB	Intact	8	100	5	71.40	12	92.3	8	61.5	n.s.
	Altered	0	0	2	28.60	1	7.7	5	38.5	
OCB	Type I (none)	4	57.10	5	71.40	8	61.5	5	38.5	n.s.
	Type II	1	14.30	1	14.30	2	15.4	4	30.8	
	Type III	0	0	0	0	0	0	1	7.7	
	Type IV	2	28.60	1	14.30	3	23.1	3	23.1	
Antibodies	LGI-1	0	0	0	0	0	0	6	46.2	*p* < 0.0001
	CASPR2							4	30.8	
	NMDAR							1	7.7	
	GAD							1	7.7	
	Zic							1	7.7	
	GFAP (pg/ml)	5,660	3,470	5,720	3,112	7,000	5,552	5,880	8,424	n.s.

*AIE, autoimmune encephalitis; BCB, blood–CSF barrier; FS, focal seizures (aware and not aware); FTBTCS, focal to bilateral tonic clonic seizures; GGE, genetic generalized epilepsy; GAS, generalized absence seizures; GMS, generalized myoclonic seizures; GTCS, generalized tonic clonic seizures, HS, hippocampal sclerosis; OCB, oligoclonal bands; OCB type II, OCBs in CSF only; OCB type III, identical OCBs in CSF and serum with additional IgG OCB in CSF; OCB type IV, identical OCB in CSF and serum; PNES, psychogenic, non-epileptic seizures; χ^2^, chi-square test*.

**Table 2:** Clinical characteristics (metric).

		**PNES (*n* = 8)**		**GGE (*n* = 7)**		**HS (*n* = 13)**		**AIE (*n* = 13)**		**Significance**
		**Median**	**IQR**	**Median**	**IQR**	**Median**	**IQR**	**Median**	**IQR**	**KW**
Years	Age	43.5	25.5	29	21	32	23.5	64	23	*p* = 0.0014
	Disease duration	4.5	6.5	8	8	14	15	1	2.5	*p* < 0.0001
Serum	Tau, pg/ml	0.91	2.19	0.44	0.57	0,47	1.07	0.83	0,84	n.s.
	NFL, pg/ml	11.18	6.98	7.54	5.63	9.87	5.86	25	24.25	*p* = 0.0009
	UCHL-1, pg/ml	8.05	13.65	8.65	18.26	8.11	10.13	24.86	23.82	*p* = 0.0033
	GFAP, pg/ml	95.85	37.55	71.6	50.5	60	120.45	92.5	100.55	n.s.
CSF	Cells, /μl	1	1.75	1	1	1	1	2	3.5	n.s.
	Protein, mg/dl	380.2	131.62	319.4	312.6	341.4	140.65	435.9	193.1	n.s.
	Tau, pg/ml	42.76	33.77	33.04	49.92	39.6	33–42	60.4	72.1	n.s.
	NFL, pg/ml	281.76	185.59	254.62	193.84	548	374.4	912	1,148	*p* = 0.0019
	UCHL-1, pg/ml	743.2	467.2	654.4	420	946.4	438.6	792.8	522	n.s.
	GFAP, pg/ml	5,660	3,470	5,720	3,112	7,000	5,552	5,880	8,424	n.s.

*AIE, autoimmune encephalitis; CSF, cerebrospinal fluid; HS, hippocampal sclerosis; IQR, interquartile range; PNES, psychogenic, non-epileptic seizures; KW, Kruskall–Wallis test with Dunn's post-hoc analysis*.

Patients with AIE were significantly older than TLE or GGE patients (*p* = 0.0014), and their disease duration before their LP was significantly shorter (*p* < 0.0001). HS and AIE patients had higher rates of cognitive alterations such as memory, executive, or other mental status disturbances then PNES and GGE patients (*p* < 0.0001), while emotional alterations such as depression and anxiety were found in all four groups. There were no significant differences in the distribution of sexes nor in the occurrence of oligoclonal bands and blood–CSF barrier (BCB) compromise. There was no statistically significant difference in CSF cell count or protein levels.

**Figure 1:**
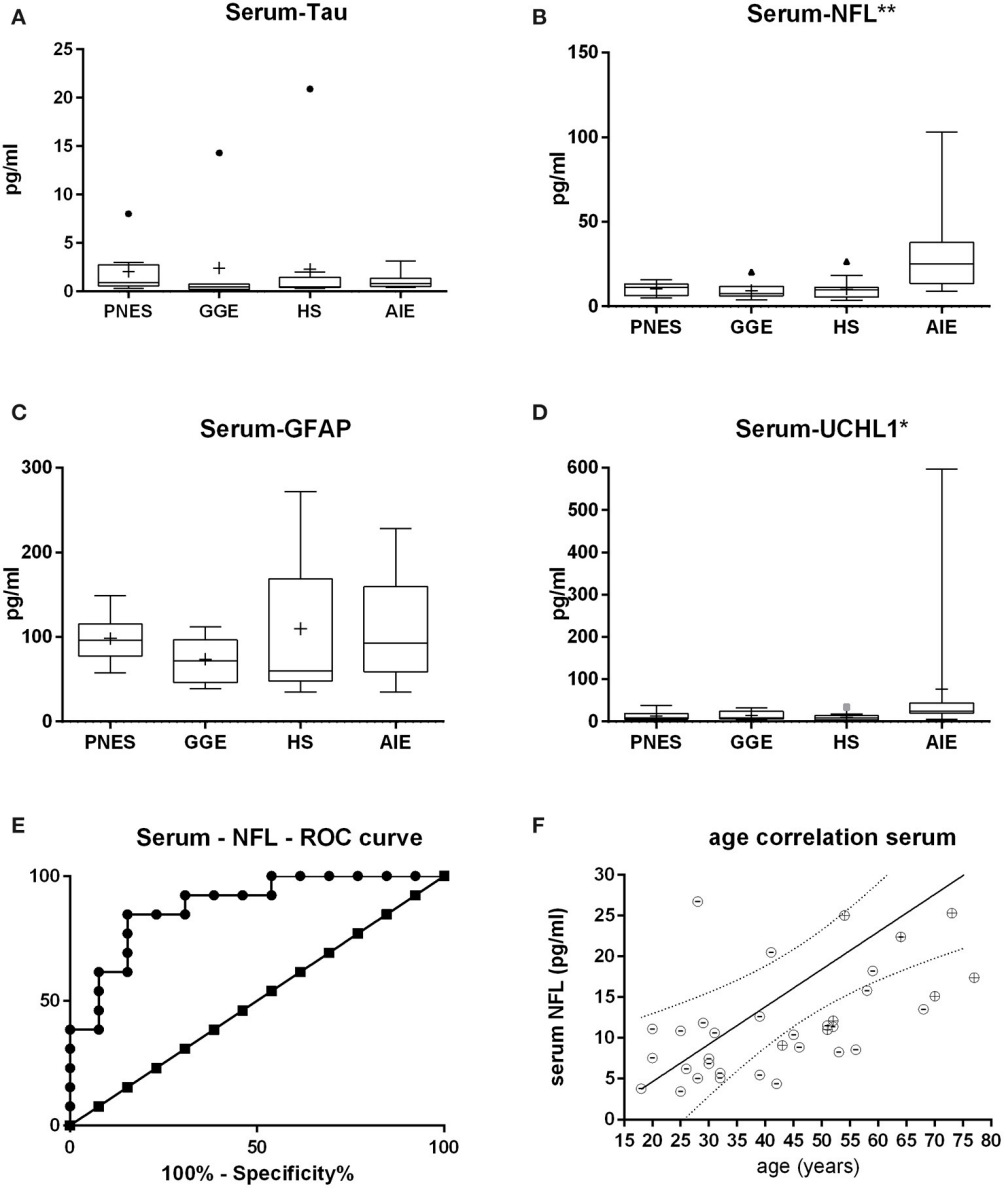
Serum levels of tau **(A)**, neurofilament (NFL) **(B)**, glial fibrillary acid protein **(C)**, and ubiquitin-carboxy-terminal hydrolase L1 **(D)**. Only NFL shows both a statistically significant and biologically robust difference in between groups since the difference in UCHL1 is mostly due to outliers. At first glance, the receiver operating characteristic curve **(E)** indicates that serum NFL may be of decent value in the question on whether epilepsy might be autoimmune-related. Of note is the fact that this may likely be a result of age alone since age strongly correlates with NFL levels both in AIE (“+ dots”) as in other etiologies (“-dots”) **(F)**. *p < 0.05, **p < 0.001.

**Figure 2:**
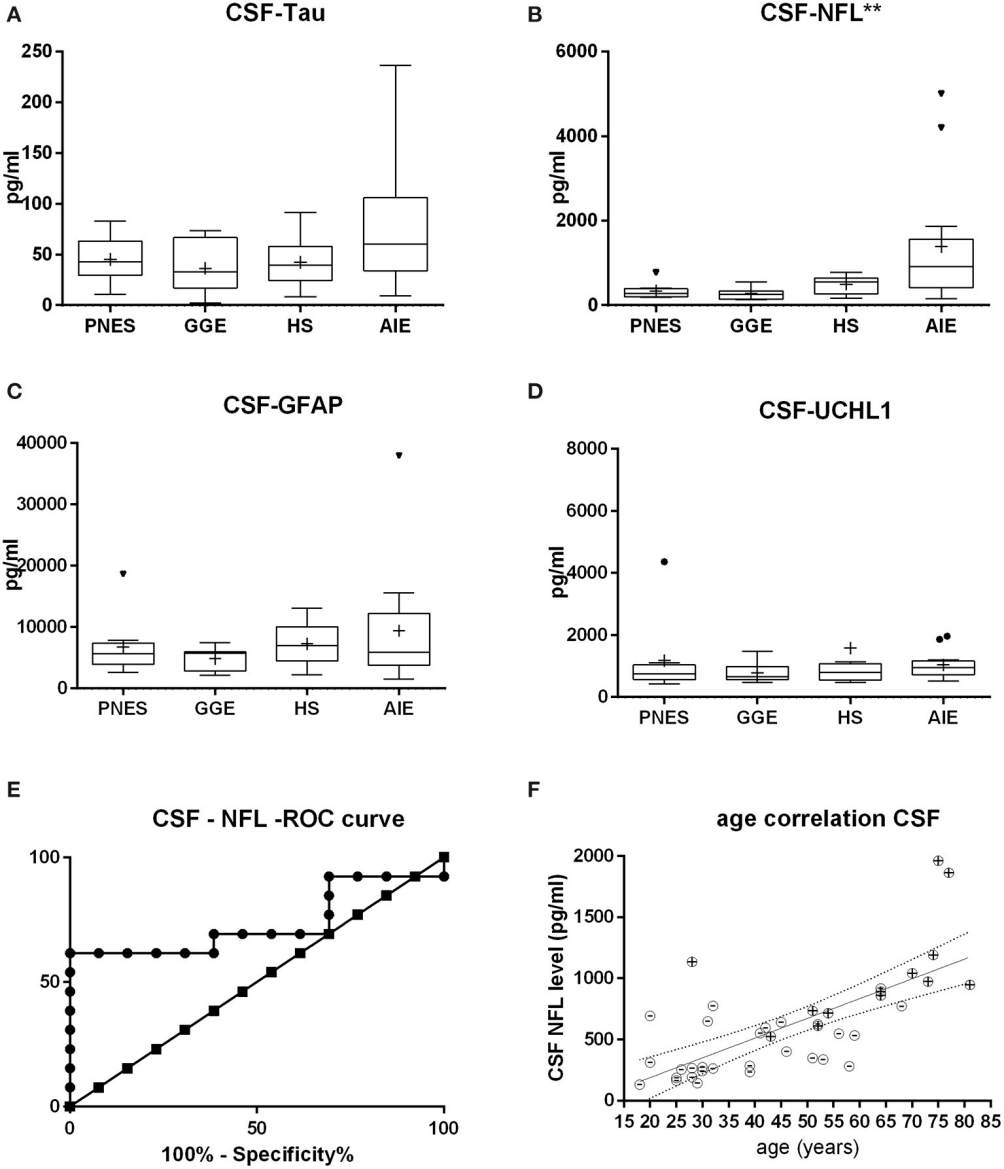
Cerebrospinal fluid (CSF) levels of tau **(A)**, neurofilament (NFL) **(B)**, glial fibrillary acid protein **(C)**, and ubiquitin-carboxy-terminal hydrolase L1 **(D)**. NFL shows the most statistically significant difference in between groups, followed by tau. At first glance, the receiver operating characteristic curve **(E)** indicates that CSF NFL may be of decent value in the question on whether epilepsy might be autoimmune-related. Of note is the fact that this may likely be a result of age alone since age strongly correlates with NFL levels both in AIE (“+ dots”) as in other etiologies (“-dots”) **(F)**. *p < 0.05, **p < 0.001.

Neither serum nor CSF tau and GFAP were different between groups, while serum UCHL-1 was just slightly but significantly higher in the AIE than in other groups (*p* = 0.0033; Figures 1, 2). This was however mostly due to outliers in the AIE group, which makes the biological significance questionable.

Serum and CSF NFL was significantly higher in AIE (median = 25; interquartile range = 24.25) than in any other group, which had half or less the median NFL levels than AIE (Figures 1, 2). NFL was positively correlated with age across all groups both in serum (Spearman *r* = 0.6; *p* < 0.0001) and CSF (Spearman *r* = 0.72, *p* < 0.0001), even if the AIE cases were excluded (Spearman *r* = 0.46 and *p* = 0.0134 in serum and *r* = 0.57 and *p* = 0.014 in CSF). Serum and CSF NFL correlated with each other as well (Spearman *r* = 0.6; *p* < 0.0001) in all groups.

ROC analysis comparing serum NFL in AIE with NFL in HS showed an area under the curve (AUC) of 0.88 (Figures 1, 2). With a cutoff value of 11.75 pg/ml, sensitivity was 84.6% (54.5–99.1%), and specificity was 84.6% (54.55–98.08%). NFL in CSF had an AUC of 0.74. A cutoff value of 600 pg/ml had a sensitivity of 69.2% (38.6–90.9%) and a specificity of 61.5% (31.6–86.1%). This result suggests serum NFL to have good discriminatory value between HS and AIE.

## Discussion

This is the first study that compared ultra-high-sensitive serum and CSF markers of brain cell damage among different seizure disorders but not the first that investigated the subject in autoimmune encephalitis or epilepsy *per se*. Parallel to our own work, NFL was shown to be elevated in untreated, autoimmune encephalitis with various antibodies and to normalize when treated with immunosuppressants (9–12). Constaninescu et al. (12) reported CSF-NFL and CSF-tau ELISA to be increased in acute autoimmune encephalitis and a correlation of the NFL levels with 1 year disability levels. The investigated patients were sicker than ours, with status epilepticus (SE) and intensive care treatment in the majority of cases. Only 50% of the 25 patients had defined ABs. In contrast, our AIE patients with defined ABs were all ambulatory. The lack of tau increments in our study may hint at different mechanisms of cell damage, i.e., additional damage due to ongoing seizures in SE. Li et al. (10) compared anti-NMDAR-encephalitis with viral meningoencephalitis and healthy controls. They found the CSF-NFL ELISA levels in anti-NMDAR-encephalitis to be higher than in viral meningoencephalitis. NFL levels correlated with cytokine levels (10). Mariotto et al. (9), who used the same SIMOA technique as we did, found NFL to be elevated in various AB-mediated encephalitis cases and to be correlated with age and outcomes. Disease progression went along with NFL elevations, while improvement went along with falling NFL levels. They found a correlation of NFL with rising age, which was very similar to the ones we report here. Körtevelyessy et al. (11) described elevated CSF-NFL and CSF-tau ELISA levels in AB-mediated encephalitis patients. Tau, in particular, was associated with the development of HS later on. The authors did not find significant associations between the levels of NFL or other biomarkers and classical markers of neuroinflammations such as CSF cell count or MRI alterations (10–13). Ouédrago et al. (14) very recently showed elevated NFL levels in drug-resistant epilepsy (DRE) when compared to well-controlled epilepsy (WCE) or healthy controls (HC). While their absolute levels were similar to our study, the age-dependent slope of NFL levels was steeper in DRE than in WCE and HC. The authors linked this finding to the expanded inflammatory CD4-T cells and proinflammatory cytokine levels, which may, in the long run, drive neurodegeneration in people with DRE (14). We recently published data on the impact of acute, tonic clonic seizures (TCS) in patients with DRE on the biomarkers described here. NFL was hardly affected, while GFAP and tau did rise more substantially in the minutes directly after a TCS. Of note is the fact that elevations of the brain cell damage biomarkers did correlate with peripheral leukocytosis (15).

The lack of classic CSF inflammatory markers found in our group such as pleocytosis, BCB compromise, or oligoclonal bands is not unusual, especially since the main AB in our sample were LGI1 and CASPR2 (16).

Of note is the fact that SIMOA-measured NFL in normally aging people seems to be in the same range as in our AIE group (17, 18). If AIE patients indeed lack NFL elevations beyond those found in normal aging, this would still merit an important result: AIE may not, *per se*, be associated with rapid cell death, which would support immunomodulatory therapies even if the disease had been going on for extended time periods (“never too late”) This hypothesis would, without a doubt, need further testing.

### Limitations

The presented data are retrospective, and the sample size is low. Hence, we did refrain from correlations with other important clinical markers such as cognition, MRI findings, seizure load, antibody titers, etc. We only measured the serum and CSF biomarker levels in “clear-cut” cases of autoimmune encephalitis and excluded cases with any ambiguity. This opens the door for confirmation bias since many patients were excluded. The strong correlation of NFL with age and the fact that the AIE patients were significantly older do significantly compromise our findings. In fact, it is likely that there is no difference between NFL levels in normal aging and our AIE group. Unfortunately, age-matched GGE, PNES, and HS were missing in this retrospective design.

## Conclusions

Our data showed elevated levels of NFL in AIE patients, while tau, GFAP, and UCHL1 had far less impressive differences between groups. At first glance, NFL seems most suitable to study AIE. At a closer look, the difference between NFL levels may be entirely attributable to aging alone. At this point, NFL testing to differentiate AIE from other types of epilepsy at a group or individual level cannot be recommended. A longitudinal analysis of NFL measurements in AIE in comparison to healthy, age-matched controls and the relation of NFL levels to treatment responses would help to determine whether such measurements could be of help in individual patients, i.e., choosing whether to continue or taper immunosuppression. Future studies should investigate whether NFL and other markers are of prospective diagnostic or prognostic value. If larger, multicentric evaluations expand the existing research, NFL in serum and/or CSF may yet become a suitable biomarker for disease activity and differentiation between AIE and other seizure disorders.

## Data Availability Statement

The raw data supporting the conclusions of this article will be made available by the authors, without undue reservation.

## Ethics Statement

The studies involving human participants were reviewed and approved by Ethical Commission of University Hospital Bonn (222/16). The patients/participants provided their written informed consent to participate in this study.

## Author Contributions

RN designed and conceptualized the study, recruited patients, personally took many blood and CSF samples, analyzed the data, and drafted the manuscript for intellectual content. KA had a major role in the acquisition of data (e.g., SIMOA measurements), designed and conceptualized the study, analyzed the data, and drafted the manuscript for intellectual content. KD searched patient files for important clinical and demographic details. CE and HR established and organized the collaboration effort, interpreted the data, and revised the manuscript for intellectual content. TZ designed and conceptualized the study, analyzed the data, and drafted the manuscript for intellectual content. RS organized the collection of CSF database, designed and conceptualized the study, analyzed the data, and drafted the manuscript for intellectual content. All authors contributed to the article and approved the submitted version.

## Conflict of Interest

RN has received fees as speaker from Eisai. KA received personal compensation for oral presentation and consulting service from Biogen Idec, Merck, Sanofi, and Roche. CE has served as a paid consultant for Desitin, Pfizer, and UCB Pharma. He was an employee of the Life and Brain Institute Bonn. HR was acting on advisory boards, gave lectures, and received research grants from Abbott, Abbvie, Bayer Health Care, Bial, Boehringer/Ingelheim, Brittania, Cephalon, Desitin, GSK, Lundbeck, Medtronic, Merck-Serono, Novartis, Orion, Pfizer, TEVA, UCB Pharma, Valeant, and Zambon. TZ received personal compensation from Almirall Biogen, Bayer, Celgene, Novartis, Roche, Sanofi, and Teva for consulting services and received additional financial support for research activities from BAT, Biogen, Novartis, Roche, Teva, and Sanofi Aventis. RS has received fees as speaker or consultant from Bial, Cyberonics, Desitin, EISAI, LivaNova, Novartis, and UCB Pharma. The remaining author declares that the research was conducted in the absence of any commercial or financial relationships that could be construed as a potential conflict of interest.
